# Unusual and delayed presentation of chronic uterine inversion in a young woman as a result of negligence by an untrained birth attendant: a case report

**DOI:** 10.1186/s13256-020-02466-x

**Published:** 2020-09-08

**Authors:** Priyanka Garg, Romi Bansal

**Affiliations:** 1 Department of Obstetrics and Gynecology, All India Institute of Medical Sciences, Bathinda, India; 2 Department of Obstetrics and Gynecology, Adesh Institute of Medical Sciences and Research, Bathinda, Punjab India

**Keywords:** Chronic uterine inversion, Haultain’s method, Parturition, Postpartum hemorrhage

## Abstract

**Background:**

Uterine inversion is a rare but known complication following parturition and may prove fatal due to neurogenic shock or postpartum hemorrhage if not corrected immediately. The incidence is variable, occurring in 1 in 2000 to 1 in 50,000 deliveries, as reported in the past. Nowadays, the incidence is declining due to better antenatal care and increasing institutional deliveries. However, in a developing country such as India, due to cultural and financial reasons, most of the deliveries are still being conducted by untrained birth attendants (“dais”) who have sparse knowledge of oxytocic drugs. Hence, proper education and training should be imparted to the traditional birth attendants and local village health practitioners about the management of labor, placental delivery, timely diagnosis, and proper management of uterine inversion to avoid this grave complication. We report this case because only a limited number of such cases have been reported so far with delayed presentation of chronic uterine inversion 8 months after delivery as a result of the negligence of an untrained birth attendant.

**Case presentation:**

We report a case of a patient with chronic uterine inversion presenting 8 months after childbirth as a result of ignorance at the time of delivery. A 22-year-old P1L1 (Para 1 Live 1) Asian woman of Punjabi ethnicity presented to our institute with a progressively increasing painless vaginal mass along with blood-stained vaginal discharge for the last 6 months and progressive dyspareunia (pain during intercourse) for the last 5 months that had worsened with time. She had experienced a full-term normal vaginal delivery at home 8 months earlier with the assistance of an untrained birth attendant (dai). Her history revealed that she had an unduly prolonged second stage of labor and was given aggressive fundal pressure due to inadequate bearing-down efforts and had collapsed after delivery but was managed conservatively by an untrained birth attendant. A provisional diagnosis of chronic uterine inversion was made on the basis of vaginal findings of a globular mass protruding from the cervix and approaching the vagina with thinning of the cervix around the mass, forming a tight constriction ring, in addition to ultrasound findings. The patient’s condition was corrected surgically using Haultain’s approach. She had a satisfactory outcome and was discharged symptom-free.

**Conclusion:**

Awareness of this complication with timely diagnosis and prompt management can significantly minimize maternal morbidity and mortality, especially in a low- and middle-income country such as India, where 70–80% of deliveries still occur in a rural setting with untrained birth attendants.

## Introduction

Uterine inversion is a rare but familiar obstetric complication after parturition and can be life-threatening if not attended to immediately [1]. It can be classified as acute, subacute, and chronic inversion, depending on its time lag from delivery. The reported incidence of uterine inversion is roughly 1 in 2000 to 1 in 50,000 births, with maternal mortality reaching up to 15% [2]. Nowadays, the incidence is declining, with only a handful of cases reported in the last decade due to better antenatal care and increasing institutional deliveries. However, in a developing country such as India, due to cultural and financial reasons, most deliveries are still conducted by untrained birth attendants (“dais”) who have sparse knowledge of oxytocic drugs. Due to its rare occurrence and lack of knowledge of birth attendants, the diagnosis is often missed at the time of delivery, especially in cases of incomplete uterine inversion due to mild or no symptoms that later advance to chronic inversion causing prolonged agony to the patient and impaired quality of life. The management approach has to be individualized, keeping in mind the type and timing of presentation and the surgeon’s caliber. Although acute uterine inversion is an emergency that can be managed nonsurgically if detected in a timely manner, chronic uterine inversion almost always requires elective surgery, either abdominally (Haultain’s or Huntington’s method) or vaginally (Spinelli’s or Kustner’s method). We report this case because only a limited number of such cases have been reported so far with delayed presentation of chronic uterine inversion at 8 months after delivery as a result of negligence by an untrained birth attendant. Hence, proper education and training should be imparted to traditional birth attendants and local village health practitioners about the management of labor, placental delivery, timely diagnosis, and proper management of uterine inversion to avoid this grave complication.

## Case presentation

**Fig. 1:**
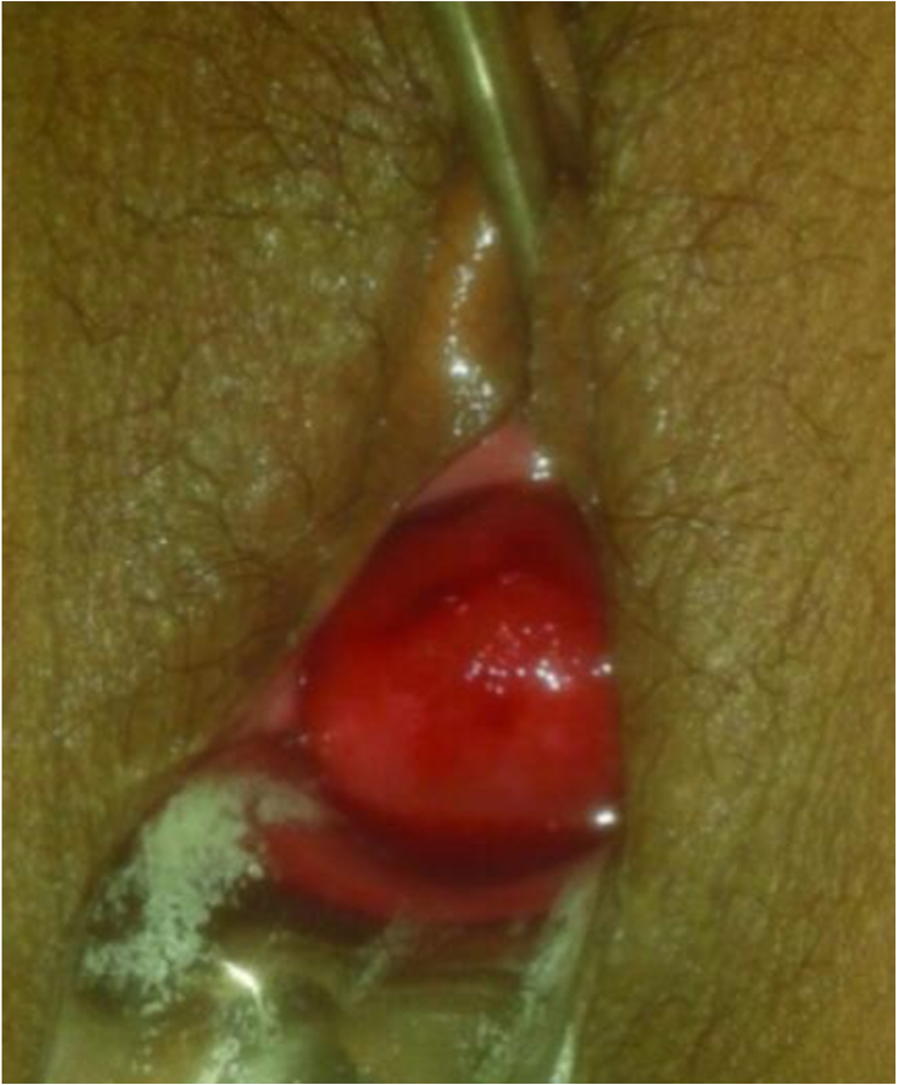
Inverted fundus of the uterus upon per speculum examination presenting as vaginal mass

We report a case of a 22-year-old Para1Live1 Asian woman of Punjabi ethnicity. The patient presented to our institution with a history of progressively increasing painless vaginal mass along with blood-stained vaginal discharge for the last 6 months and dyspareunia (pain during intercourse) for the last 5 months that had worsened with time. Although her menstrual cycles were regular with normal duration and amount, she had intermenstrual spotting on and off. She had experienced a full-term normal vaginal delivery at home 8 months earlier with the assistance of an untrained birth attendant (dai) and had delivered a normal healthy baby girl. Her history revealed that she had an unduly prolonged second stage of labor and was given aggressive fundal pressure due to inadequate bearing-down efforts and had collapsed after delivery but was managed conservatively by a village health practitioner. She was a homemaker, did not consume alcohol, and was a nonsmoker. Due to familial responsibilities, the patient did not seek help at a tertiary center until 8 months later, when she reported to our institution. Her past medical and family history was unremarkable. She had no history of any treatment in the past. Upon admission to our institute, the patient was conscious and well oriented to time, place, and person. Her general examination revealed that she was thin with moderate pallor. Her blood pressure was 110/70 mmHg; her pulse rate was 94 beats/minute; and she was afebrile to touch. Her systemic examination did not reveal any abnormality. Her abdominal examination was unremarkable. Local examination revealed a 5-cm × 4-cm congested globular mass with smooth margins that bled upon manipulation. Upon vaginal examination, a round, convex mass was palpated, originating from the cervix and reaching into the vagina, as shown in Fig. 1. A thinned-out cervical rim could be felt around the mass, forming a firm constriction ring, and uterine sound could not be passed around the mass. The uterine fundus was not appreciated. The uterus could not be felt by per rectal examination. With the patient’s history and clinical findings, a tentative diagnosis of chronic uterine inversion was made, keeping other differential diagnosis possibilities (submucous fibroid, uterine polyp) in mind, and ultrasonography (USG) was suggested. USG revealed an inverted uterus with the fundus of the uterus within the vagina. She was diagnosed with long-standing neglected chronic uterine inversion with delayed presentation. Laboratory investigations revealed hemoglobin of 8g/dl. Her other hematological and biochemical test results were normal. She was planned for elective surgery under the cover of culture-sensitive broad-spectrum antibiotics (injection linezolid 1.2 g twice daily, injection gentamicin 80 mg twice daily, and injection metronidazole 500 mg thrice daily for 2 days) and after transfusing 2 units of packed red cells.

**Fig. 2:**
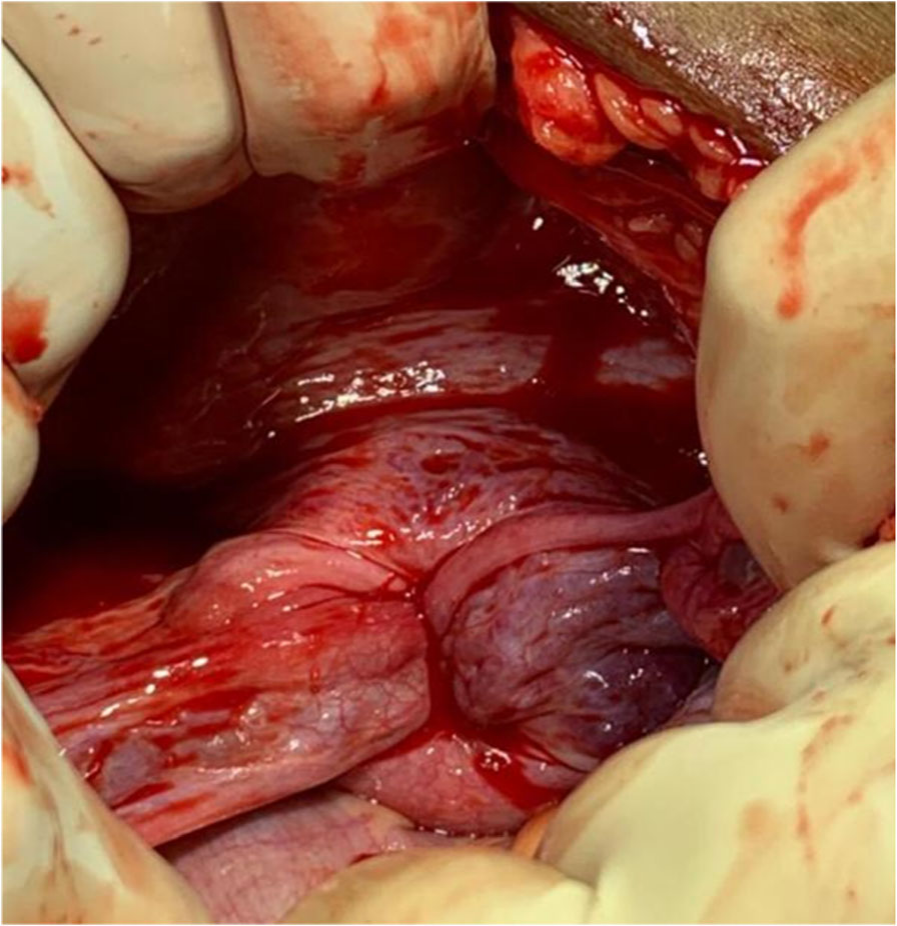
Flower vase appearance

**Fig. 3:**
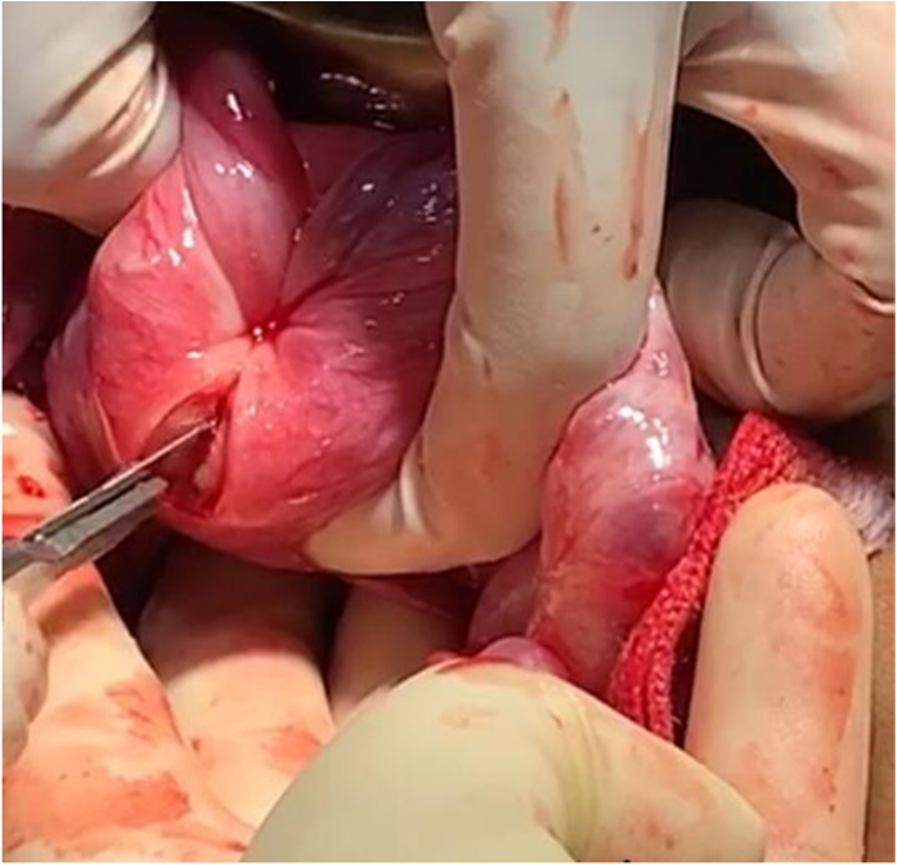
Vertical incision on the posterior wall of the uterus and constriction ring

**Fig. 4:**
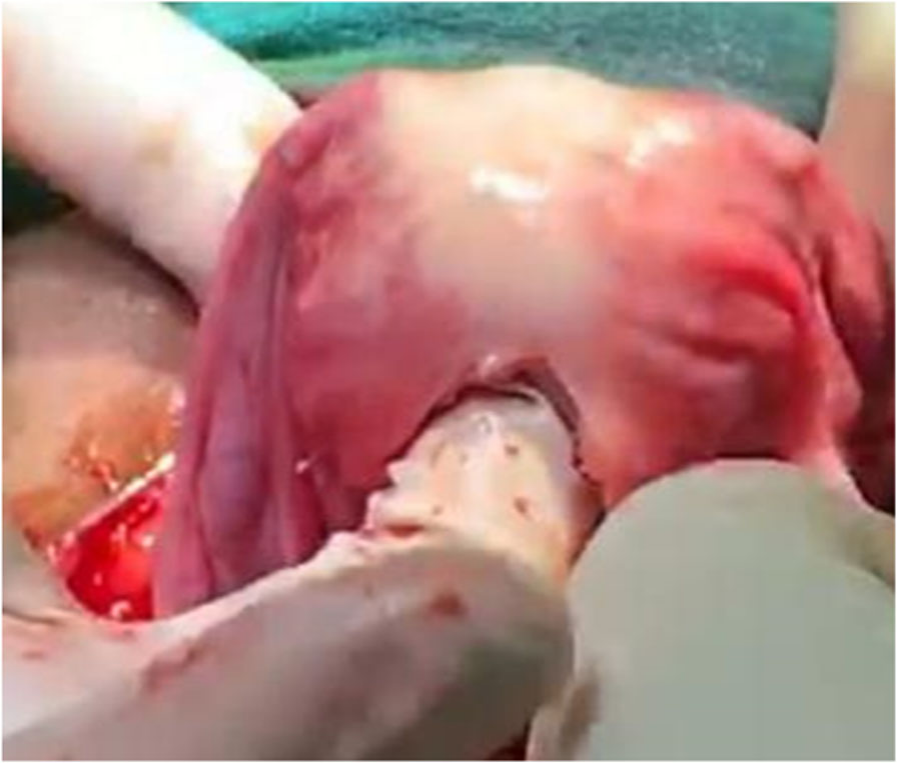
Repositioning of the uterus

Initially, under the effect of anesthesia, manual repositioning was attempted with no success. Laparotomy was then performed, which displayed a typical flower vase appearance with fundal cupping of the uterus with inward pulling of tubes and ovaries, as shown in Fig. 2, thus confirming the diagnosis of chronic uterine inversion. The incision on the posterior uterine wall at the site of the constriction ring and gentle pulling on round ligaments resulted in immediate repositioning of the uterus (Haultain’s approach), as shown in Figs. 3 and 4. Her postoperative period was uneventful, and she was discharged in satisfactory condition after 3 days on oral antibiotics (tablet linezolid 600 mg twice daily and tablet metronidazole 400 mg twice daily for 5 days).

At her follow-up visit at 6 weeks, the patient was symptom-free, and her local examination of genitalia revealed no abnormality. A USG report revealed normal position of the uterus. In later follow-up visits at 3 and 6 months, the patient reported normal menstruation and normal sexual activity.

## Discussion

We report a case of chronic uterine inversion with delayed presentation at 8 months postpartum due to mismanaged delivery at home by an untrained birth attendant (dai). Only a few such cases have been reported so far in the literature. “Uterine inversion” refers to the abnormal protrusion of the uterine fundus through the vaginal orifice so that the uterus is turned inside out. It can be complete (if it passes through the cervix) or incomplete (if it does not pass through the cervix). On the basis of its diagnosis from the time of childbirth, it can be classified as acute (within 24 hours of delivery), subacute (between 24 hours and 4 weeks), or chronic (4 weeks after delivery). Acute uterine inversion is a potentially fatal complication of a mismanaged third stage of labor, leading to severe postpartum hemorrhage and shock; if not managed aggressively, it can prove lethal. Incomplete acute uterine inversion may be overlooked due to mild or no symptoms [3] and inexperience of untrained birth attendant. It later can advance to chronic symptoms, as seen in our patient’s case. This could be the reason for the late presentation in our patient, who reported to us 8 months after delivery. A similar case was reported by Ali and Kumar who presented with chronic uterine inversion six months after mismanaged delivery at home by an untrained birth attendant [2]. The likely cause in our patient could have been aggressive fundal pressure in the second stage of labor due to inadequate bearing-down efforts, short umbilical cord, and excessive traction on the cord before the signs of placental separation [3], as suggested by the history given by the patient. The inversion could have been incomplete at that time, which later advanced to chronic inversion with time.

The patient usually presents with menometrorrhagia, foul-smelling discharge per vaginum, or a mass coming out of the vagina (as seen in our patient’s case). Other accompanying symptoms can be pain in the lower abdomen or history of postpartum shock, which can be neurogenic or hemorrhagic. (Our patient probably had neurogenic shock.) Clinical examination usually reveals a globular mass in the vagina that can be congested, hyperemic, ulcerated, or infected. It has to be differentiated from other causes, such as submucous myoma, endometrial polyp, cervical carcinoma, or uterovaginal prolapse. USG and magnetic resonance imaging are helpful whenever there is a diagnostic dilemma.

The management of acute uterine inversion includes treatment of hypovolemic shock and immediate repositioning of the uterus, either manually or by hydrostatic pressure (Sullivan’s method). However, chronic inversion requires a surgical approach because the chronically inverted uterine walls have meager resilience due to complete involution. The firmness of the constriction ring and the inelastic walls have to be subdued along with the rigidity of the retaining myometrium, which cannot be overcome [4]. The goal is to divide the cervical constriction ring either anteriorly or posteriorly [3]. The abdominal methods are Huntington’s and Haultain’s methods (used in our patient’s case). In Huntington’s method, bilateral round ligaments and the uterus below the inversion site are grasped with Allis tissue forceps and gently pulled upward until the uterus returns to its original position. In Haultain’s method, a longitudinal incision is made over the cervical ring posteriorly and with gentle traction, and the uterus is reinverted, followed by suturing of the uterus in two or three layers [5]. The vaginal surgeries include Spinelli’s and Kustner’s techniques for repositioning the prolapsed fundus through the anterior and posterior transections (through cul-de-sac), respectively [2]. The abdominal route is preferred due to better vision, precise incision of the constriction ring, easy repositioning with traction on the round and broad ligaments, adequate hemostasis, and flawless suturing over the vaginal route [6]. In subsequent pregnancies, recurrence is rare if good obstetrical care is provided, as reported by previous studies as well [4, 7]. Sinha and Sinha reported five cases of chronic uterine inversion that were successfully repaired by using the conventional Haultain method, with three of the patients conceiving later [8]. With advancements in medicine, cases of chronic uterine inversion corrected by robotic and laparoscopic surgeries have been reported with quick recovery and a fair success rate. However, robotic surgery facilities were not available at our institute, and due to financial considerations, the patient could not afford laparoscopic correction. Because our patient was desirous of future childbirth, we adopted the Haultain abdominal approach and achieved a good surgical outcome. The patient was counseled regarding the use of contraceptives for at least 2 years and to opt for elective lower-segment cesarean section in the subsequent pregnancy.

## Conclusion

Chronic uterine inversion usually occurs following parturition, which might have been missed at the time of delivery in our patient’s case. Although the incidence of uterine inversion is very low nowadays due to better antenatal care and institutional deliveries, the possibility of its occurrence cannot be negated. Awareness of this complication, especially among untrained birth attendants, may prevent improper management and such complications causing prolonged agony in the patient. Thus, timely diagnosis and immediate management can result in a significant reduction of maternal morbidity and mortality, especially in a developing country such as India, where women are usually ignorant of their health, leading to delay in seeking professional help.
